# The Court and the Hospitals

**Published:** 1889-03-30

**Authors:** 


### THE COURT AND THE HOSPITALS.

The Rev. F. O. Morris writes: I happened to see in the paper yesterday that the large bouquets of flowers taken, as usual, to the Court the day or so before, were of much enlarged size; so much so that they had to be carried in the hands of attendants outside. It came into my mind this morning, as, I hope, a " happy thought," that it would be a very good deed to have these beautiful nosegays collected together afterwards to send to the various children's hospitals and infirmaries; and I think it would be quite easy to have this done, all the various owners leaving nearly together at one time from the same place; and there might be some " reception-room," anywhere near at hand, where they might all be left for the purpose by the ladies after the ceremony of presentation is over, as they drive to their several homes.

" . . . I love the field flowers too,
Because they are a blessing given
E'en to the poorest little one
That wanders 'neath the vault of heaven;
The garden flowers are reared for few,
And to those few belong alone."

But let them thus " give of their abundance," and their gifts will leave a fragrant scent behind.

